# Larvicidal, growth inhibitory and biochemical effects of soil bacterium, *Pseudomonas* sp. EN4 against *Spodoptera litura* (Fab.) (Lepidoptera: Noctuidae)

**DOI:** 10.1186/s12866-023-02841-w

**Published:** 2023-04-03

**Authors:** Sunaina Sarkhandia, Meena Devi, Geetika Sharma, Rohit Mahajan, Pooja Chadha, Harvinder Singh Saini, Sanehdeep Kaur

**Affiliations:** 1 Department of Zoology, Guru Nanak Dev University, Punjab, Amritsar, 143005 India; 2 Department of Microbiology, Guru Nanak Dev University, Punjab, Amritsar, 143005 India

**Keywords:** *Spodoptera litura*, *Pseudomonas* sp., Insecticidal potential, Nutritional physiology, Genotoxicity, Biochemical analysis

## Abstract

**Background:**

*Spodoptera litura* (Fabricius) (Lepidoptera: Noctuidae) also known as tobacco caterpillar, is one of the most serious polyphagous pests that cause economic losses to a variety of commercially important agricultural crops. Over the past few years, many conventional insecticides have been used to control this pest. However, the indiscriminate use of these chemicals has led to development of insecticide resistant populations of *S. litura* in addition to harmful effects on environment. Due to these ill effects, the emphasis is being laid on alternative eco-friendly control measures. Microbial control is one of the important components of integrated pest management. Thus, in search for novel biocontrol agents, the current work was carried out with the aim to evaluate the insecticidal potential of soil bacteria against *S. litura*.

**Results:**

Among the tested soil bacterial isolates (EN1, EN2, AA5, EN4 and R1), maximum mortality (74%) was exhibited by *Pseudomonas* sp. (EN4). The larval mortality rate increased in a dose-dependent manner. Bacterial infection also significantly delayed the larval development, reduced adult emergence, and induced morphological deformities in adults of *S*. *litura*. Adverse effects were also detected on various nutritional parameters. The infected larvae showed a significant decrease in relative growth and consumption rate as well as efficiency of conversion of ingested and digested food to biomass. Histopathological studies indicated damage to the midgut epithelial layer of larvae due to the consumption of bacteria treated diet. The infected larvae also showed a significantly decreased level of various digestive enzymes. Furthermore, exposure to *Pseudomonas* sp. also caused DNA damage in the hemocytes of *S. litura* larvae.

**Conclusion:**

Adverse effects of *Pseudomonas* sp. EN4 on various biological parameters of *S. litura* indicate that this soil bacterial strain may be used as an effective biocontrol agent against insect pests.

## Introduction

Insect pests are a major threat to various agricultural crops throughout the world. Many of the agricultural insect pests belong to the order Lepidoptera, which is the second largest order of class Insecta. Among lepidopterans, the common cutworm, *Spodoptera litura* (Fabricius) (Lepidoptera: Noctuidae) is one of the major polyphagous pests of agricultural crops [1]. It is an economic pest in India, China and Asia–Pacific regions where it causes losses to many economically important cultivated field crops and vegetables [2]. It has been reported to damage 112 species of plants belonging to 44 families that include 40 species from India [3]. In recent years, there has been an increase in the occurrence of *S. litura* in India causing severe economic losses to commercial and vegetable crops including soybean, cabbage, cauliflower, groundnut etc. [4–6]. Approximately, 47% yield losses have been reported in groundnut due to *S. litura* in India [7]. Recently, Sahu et al. [5] documented 54% infestation of this pest on cabbage crop. *S. litura* completes a number of generations per year that occasionally overlap [8]. The females exhibit a strong migratory ability and high reproductive potential [9]. Early larval instars are gregarious and mostly scrape the soft part of leaves, while later instars fully defoliate the plants when present in large numbers, and cause significant crop losses. Chemical control is the most commonly used strategy by farmers to manage this pest.

To improve agricultural productivity, pesticides continue to be a significant input in modern agriculture. Synthetic pesticides are effective against a variety of insect species because they are cheap, easily available, fast-acting, and highly reliable. A single application can control a variety of pest species and leaves a persistent residue that kills insects for hours or even days after its application [10]. For the control of *Spodoptera* spp., a number of insecticides from various chemical classes are used, either individually or in combination. The common conventional and some new chemistry insecticides used against *S. litura* include lambda-cyhalothrin, chlorpyrifos, quinolphos, deltamethrin, cypermethrin, spinosad, abamectin, indoxacarb, emamectin benzoate, lufenuron etc. [11, 12]. However, these insecticides also result in a direct impact on human health and the environment. Besides contaminating the soil, air and water bodies, they also adversely affect the non-target organisms [13]. One of the most serious problems associated with the use of synthetic insecticides is the development of resistance in insects. Currently, high levels of resistance have been reported in various lepidopteran pests to many insecticides including organochlorines, organophosphates, carbamates and pyrethroids, [14, 15]. Due to its polyphagous nature, *S. litura* has been exposed to a number of insecticides. There are reports indicating the development of varying levels of resistance in *S. litura* to many of the conventional insecticides [16–18]. In such situations, where chemicals are causing a harmful impact on environment, the use of biopesticides has emerged as a sustainable alternative for the suppression of insect pests. Biopesticides based on pathogenic microorganisms such as fungi, bacteria and viruses are specific to a target pest offering an ecologically sound and effective solution to pest problems [19, 20].

Entomopathogenic bacteria belonging to the genus *Bacillus* such as *Bacillus cereus*, *Bacillus sphaericus*, *Bacillus popilliae*, *Bacillus subtilis*, and *Bacillus thuringiensis*, have been used against various insect pests [21, 22]. Among these, *B. thuringiensis* (*Bt*) (Berliner) is one of the most commercially exploited bacteria for insect control. It produces a crystal protein (δ-endotoxin) during bacterial sporulation that is capable of causing lysis of gut cells when consumed by susceptible insects [23, 24]. In comparison to synthetic pesticides, Bt spores and parasporal crystals are thought to be safer and more specific. *B. thuringiensis* sub-species including *B. thuringiensis* subsp. *kurstaki* and *B. thuringiensis* subsp. *aizawai*, are highly toxic to lepidopteran larval species [25]. A number of *Bt* formulations including Delfin, Halt, Biosap, Dipel, and Biobit, are commercially available in the market [26]. Shingote et al. [27] found that Vip1/Vip2 toxins of *Bt* had 60% insecticidal effectiveness against the Coleopteran stored grain pests. However, the most important threat to the continued efficacy of *Bt* insecticidal proteins (toxins) is the evolution of resistance in target pests. Alteration of toxin binding sites is one of the main mechanisms that cause resistance [28]. Recent reports documented resistance in lepidopteran pests such as *Helicoverpa zea* (Boddie) and *Plutella xylostella* (Linnaeus) against *Bt* formulations under lab and field conditions [29, 30].

Recent reports documented pathogenicity of *Burkholderia*, *Chromobacterium*, *Pseudomonas*, *Serratia*, *Streptomyces*, and *Yersinia* species against various insect pests, primarily against lepidopteran caterpillars [31]. Most entomopathogenic bacteria produce a variety of toxins with similar mechanisms of action to *Bt* [32]. Therefore, there is a stringent need to explore novel bacterial isolates having insecticidal potential.

In the current studies, we investigated soil bacterial isolates for their insecticidal potential on second instar *S. litura* larvae. Additionally, we examined the effect of selected bacterial strains on the nutritional, biochemical, histopathological and genotoxic parameters of *S. litura*.

## Results

### Screening bioassay

**Fig. 1:**
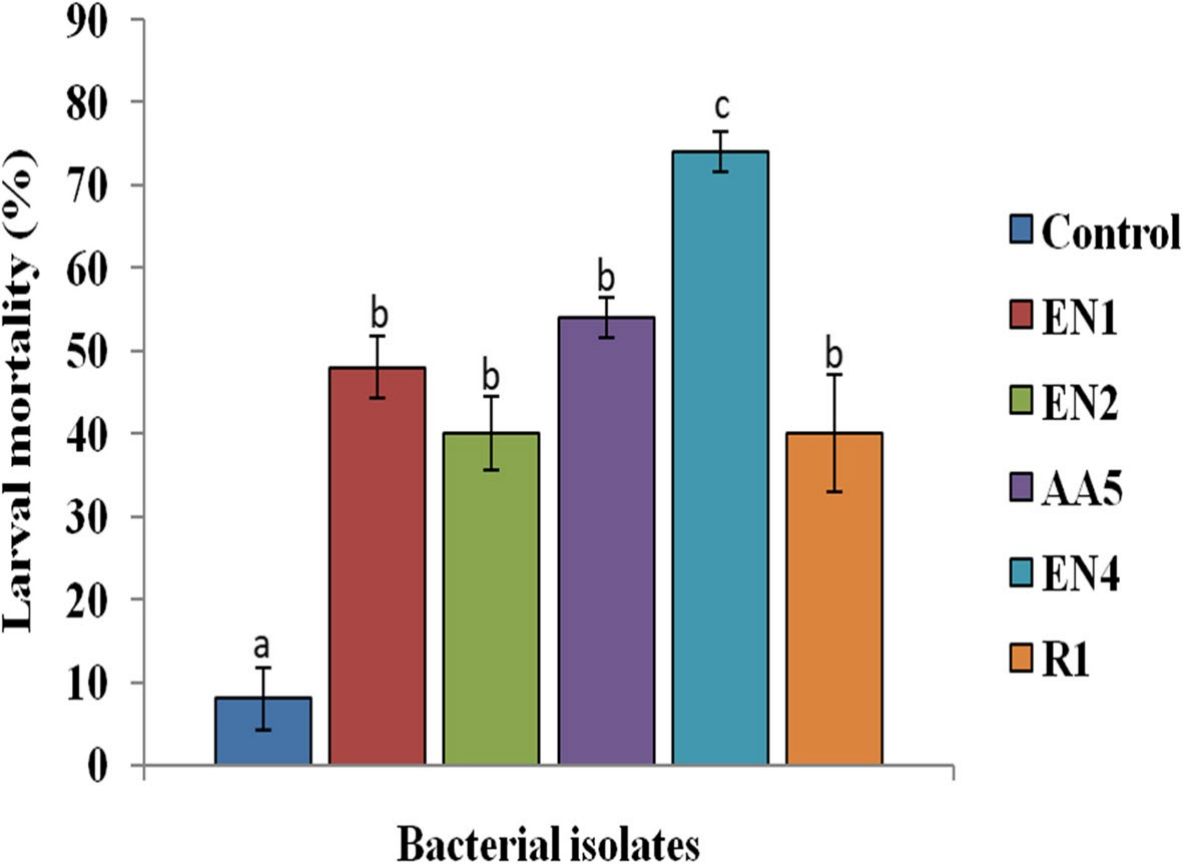
Screening of different bacterial isolates for insecticidal potential against second instar *S. litura* larvae at 1.8 × 10^9^ cfu/ml (approx). Bars represent the Mean ± SE. Different letters above the bars represent significant differences at Tukey’s test *P* ≤ 0.05

All the bacterial isolates induced significantly higher larval mortality than control (Fig. 1). Among the tested cultures, EN4 induced the highest mortality i.e. 74%. As per the biochemical/microbiological analysis, the EN4 bacterial isolate was observed to be rod shaped, gram-negative, aerobic and non-pigmented bacterium. It was identified as *Pseudomonas* sp. EN4 (GenBank accession number MW678603) [33]. It was observed that the *Pseudomonas* sp. was closely related to the members of the genus *Pseudomonas* and showed 98% nucleotide identity with *Pseudomonas citronellolis* strain NBRC 103,043 (NR114194).

### Concentration response test

**Fig. 2:**
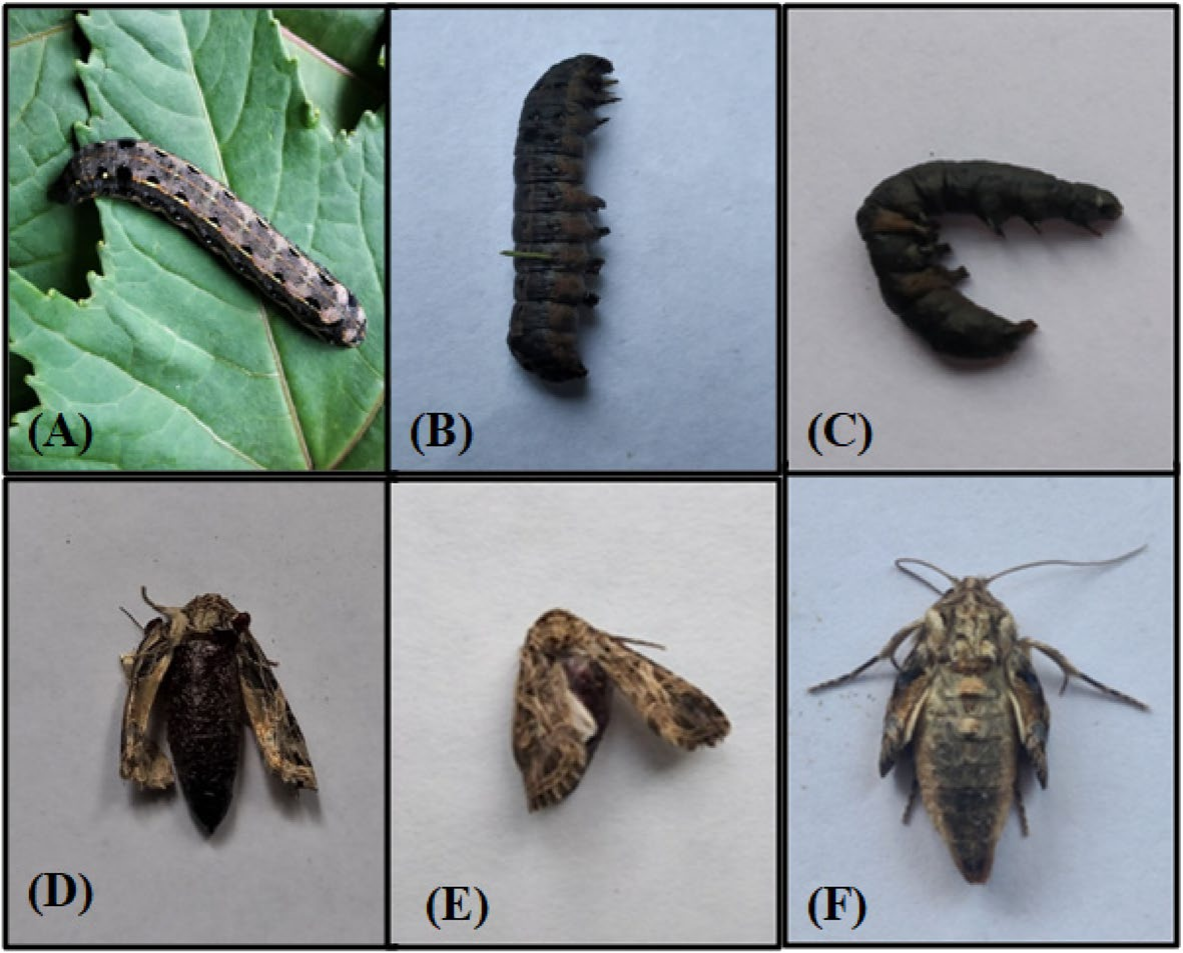
**A** Healthy larva, **B** and **C** Dead larvae, **D**-**F** Morphologically deformed adults

The concentration response assay results showed that *Pseudomonas* sp. induced toxic effects on various biological parameters of *S. litura* when ingested orally. With respect to 6% in control group, the larval mortality ranged from 42 to 78% in treated larvae (F = 25.25, *p* ≤ 0.05) (Table 1). It was a dose-dependent effect. At higher concentrations (3.4 × 10^7^ and 4.3 × 10^9^ cfu/ml), larval mortality started after 3 days of treatment and continued till 15 days. Compared to healthy larvae, the infected larvae became lethargic, stopped feeding, and their bodies eventually turned black leading to death (Fig. 2A, B, C). The LC_50_ and LC_90_ values of *Pseudomonas* sp. against *S. litura* larvae were found to be 1.21 × 10^9^ cfu/ml (95% confidence interval: 0.93–1.41 × 10^9^ cfu/ml) and 5.23 × 10^9^ cfu/ml (95% confidence interval: 3.90–9.18 × 10^9^ cfu/ml) using Probit analysis.

**Table 1:** Effect of different concentrations of *Pseudomonas* sp. on development and adult emergence of *S. litura*

Concentrations (cfu/ml)	Larval period (in days)	Pupal period (in days)	Total development period (in days)	Larval mortality (%)	Adult emergence (%)	Adult deformities (%)
Control	10.21 ± 0.45^a^	9.58 ± 0.67^ab^	19.77 ± 0.82^a^	6.0 ± 4.0^a^	95.52 ± 2.74^c^	2.84 ± 2.84^a^
1.2 × 10^3^	11.76 ± 0.57^ab^	9.22 ± 0.46^ab^	20.98 ± 0.67^a^	42.0 ± 10.19^b^	77.82 ± 6.54^bc^	11.66 ± 7.25^a^
1.9 × 10^5^	13.54 ± 0.44^bc^	8.30 ± 0.38^ab^	21.84 ± 0.20^ab^	58.0 ± 3.74^bc^	58.98 ± 6.98^ab^	16.66 ± 10.53^ab^
2.6 × 10^6^	13.20 ± 0.48^bc^	8.40 ± 0.50^ab^	21.60 ± 0.67^a^	70.0 ± 3.16^c^	48.30 ± 8.48^ab^	20.0 ± 12.24^ab^
3.4 × 10^7^	14.12 ± 0.56^c^	8.0 ± 0.63^a^	22.12 ± 1.14^ab^	72.0 ± 3.74^c^	38.32 ± 5.0^a^	40.0 ± 24.49^ab^
4.3 × 10^9^	14.60 ± 0.40^c^	10.40 ± 0.40^b^	25.0 ± 0.44^b^	78.0 ± 3.74^c^	33.32 ± 9.12^a^	80.0 ± 20.0^b^
F-value	11.06**	3.07*	5.72**	25.25**	12.40**	3.57*

Figures are Mean ± Standard Error. Means followed by different superscript letters (a, b, c) within a column are significantly different. Tukey’s test *P* ≤ 0.05, ** Significant at 1%, * Significant at 5%

Oral ingestion of *Pseudomonas* sp. significantly influenced the growth and development of *S. litura* larvae. Except for the lowest concentration, all the other concentrations of *Pseudomonas* sp. extended the larval period significantly by 3.33 to 4.39 days with respect to control (F ꞊ 11.06, *p* ≤ 0.05). A significant effect was also detected on the pupal period. With respect to control the total development period increased significantly by 5.23 days at the highest concentration (F = 5.72, *p* ≤ 0.05) (Table 1).

The results also revealed a decreasing trend in adult emergence due to bacterial infection with a significant effect at the higher concentrations of *Pseudomonas* sp. (F ꞊ 12.40, *p* ≤ 0.05). The bacterial infection also induced morphological deformities such as crumpled and underdeveloped wings of adults (Fig. 2D, E, F). As very few adults emerged at the higher concentrations, no data could be recorded on reproductive potential.

### Nutritional assay

Food utilization analysis showed that *Pseudomonas* sp. negatively affected all the nutritional parameters viz. RGR, RCR, ECI, ECD and AD of *S. litura* larvae (Table 2). Consumption of higher concentrations of bacterial cell suspension led to a significant decrease in the relative growth and consumption rate of larvae. With respect to control, the values of RGR and RCR dropped by 13.33 to 50% and 12.49 to 22.58%, respectively. The results presented in Table 2, also depicted a significant negative impact on ECI and ECD values especially at higher concentrations (ECI: F ꞊ 43.65, *p* ≤ 0.05, ECD: F = 58.44, *p* ≤ 0.05). All the concentrations of *Pseudomonas* sp. also significantly decreased the approximate digestibility of food (F ꞊ 59.94, *p* ≤ 0.05).

**Table 2:** Effect of different concentrations of *Pseudomonas* sp. on nutritional parameters of *S. litura*

Concentrations (cfu/ml)	RGR (mg mg^−1^ d^-^^1^)	RCR (mg mg^−1^ d^−1^)	ECI (%)	ECD (%)	AD (%)
Control	0.30 ± 0.001^e^	14.17 ± 0.08^c^	2.15 ± 0.02^d^	30.03 ± 2.10^e^	92.40 ± 0.37^d^
1.2 × 10^3^	0.28 ± 0.005^de^	13.78 ± 0.39^c^	2.08 ± 0.02^d^	19.69 ± 1.35^d^	84.80 ± 0.79^c^
1.9 × 10^5^	0.26 ± 0.006^d^	13.39 ± 0.36^bc^	1.97 ± 0.03^ cd^	14.89 ± 1.19^ cd^	82.0 ± 0.56^bc^
2.6 × 10^6^	0.23 ± 0.007^c^	12.98 ± 0.35^bc^	1.80 ± 0.01^c^	10.52 ± 1.13^bc^	80.70 ± 0.73^bc^
3.4 × 10^7^	0.19 ± 0.008^b^	12.40 ± 0.18^b^	1.56 ± 0.07^b^	6.29 ± 0.59^ab^	79.60 ± 1.57^b^
4.3 × 10^9^	0.15 ± 0.005^a^	10.97 ± 0.24^a^	1.26 ± 0.08^a^	3.96 ± 0.39^a^	69.40 ± 1.22^a^
F- value	80.39**	15.29**	43.65**	58.44**	59.94**

Figures are Mean ± Standard Error. Means followed by different superscript letters (a, b, c, d, e) within a column are significantly different. Tukey’s test *P* ≤ 0.05. **Significant at1%

### Histopathological analysis

**Fig. 3:**
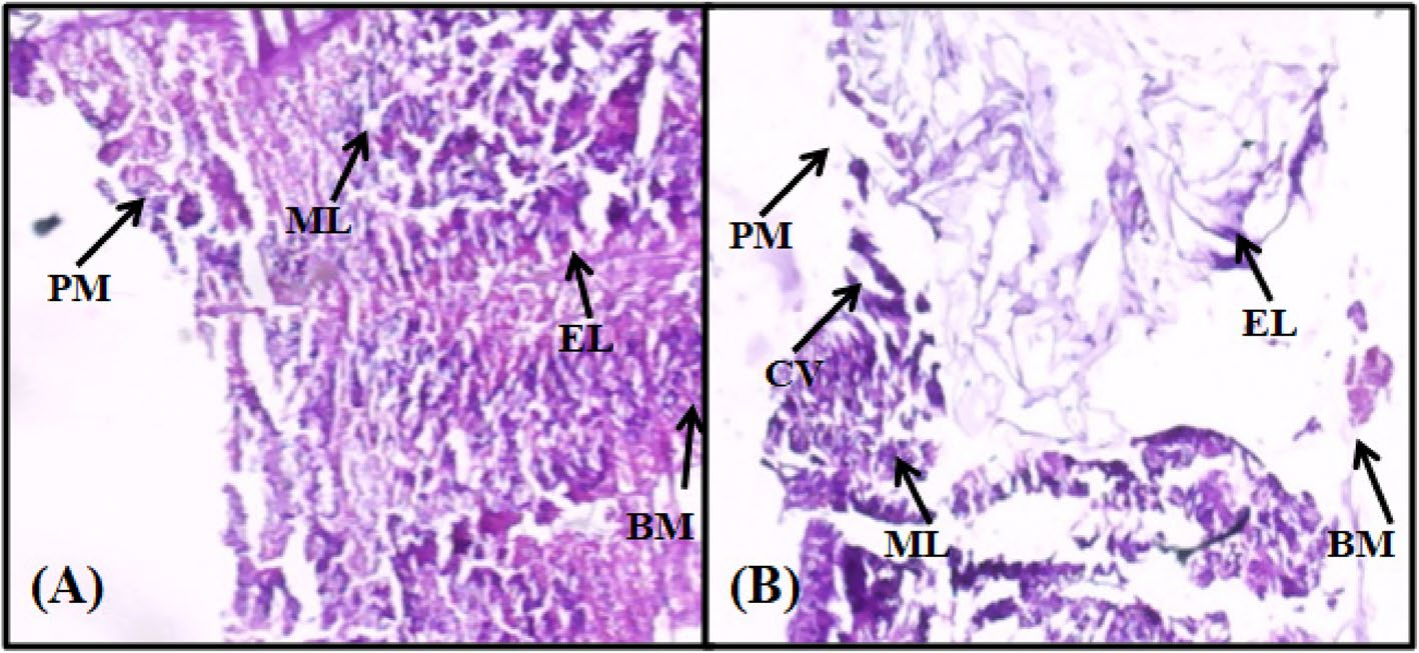
Longitudinal sections of midgut tissue from control and treated *S. litura* larvae (400x) after 96 h of treatment. **A** Midgut of control larvae showing intact basement membrane (BM), no disruption in epithelial layer (EL), peritrophic membrane (PM) and muscle layers (ML). **B** Midgut of larvae treated with *Pseudomonas* sp. showing degradation in BM, EL, PM, ML, and prominent cytoplasmic vacuoles (CV)

*S. litura* larvae from the control group displayed a well-conserved layer of muscles and epithelial cells (Fig. 3A). Infection due to *Pseudomonas* sp. caused morphological and cellular damage to epithelial, peritrophic, basement membrane and muscle layers of midgut tissue of *S. litura*. Most of the cells appeared enlarged and disorganized with prominent cytoplasmic cavities. Detachment of the epithelial layer from the basement membrane was also detected (Fig. 3B).

### Assessment for the presence of bacteria in larval hemolymph

The growth of *Pseudomonas* sp. was observed in the hemolymph of treated *S. litura* larvae while no growth of *Pseudomonas* sp. was detected in control larvae.

### Biochemical analysis

The data presented in Table 3, highlights the inhibitory effect of *Pseudomonas* sp. on the activities of digestive enzymes of *S. litura*. When the larvae were allowed to feed on *Pseudomonas* sp. treated leaves, a significant decrease of 34.64 and 74.28% was detected in α-amylases activity after 48 and 96 h, respectively, compared to the control. Similarly, bacterial treatment suppressed the activity of α-glucosidases with a significant drop of 78.71% after 96 h with respect to control. A similar trend was observed in the case of ß-glucosidases. *Pseudomonas* sp. also influenced the level of α and ß-galactosidases which showed 20.4 and 19.76 times less activity 96 h post-treatment with respect to control larvae. As is evident from Table 3, significant drop was also observed in activities of lipases and proteases 48 and 96 h post-treatment of *Pseudomonas* sp.

**Table 3:** Effect of *Pseudomonas* sp. (LC_50_ values) on the activity of digestive enzymes of third instar larvae of *S*. *litura*

Enzymes (µM/mg)	Treatments	Time Intervals	
		**48 h**	**96 h**
**α-Amylase**	**Control**	30.28 ± 1.04	42.73 ± 1.08
	**EN4**	19.79 ± 1.03	10.99 ± 1.57
**‘t’-value**		5.03*	15.80**
**α- Glucosidases**	**Control**	15.42 ± 0.95	19.59 ± 0.56
	**EN4**	9.53 ± 1.14	4.17 ± 0.30
**‘t’-value**		4.42*	41.52**
**ß- Glucosidases**	**Control**	18.06 ± 0.62	26.92 ± 0.66
	**EN4**	8.58 ± 0.15	4.59 ± 1.38
**‘t’-value**		13.57**	16.07**
**α- Galactosidases**	**Control**	18.15 ± 0.64	23.61 ± 0.46
	**EN4**	8.67 ± 0.23	3.21 ± 0.30
**‘t’-value**		11.11**	49.75**
**ß- Galactosidases**	**Control**	18.24 ± 0.46	22.62 ± 0.32
	**EN4**	6.88 ± 0.69	2.86 ± 0.23
**‘t’-value**		9.76*	50.70**
**Lipases**	**Control**	20.13 ± 0.52	26.78 ± 0.45
	**EN4**	11.01 ± 1.23	4.16 ± 0.74
**‘t’-value**		5.27*	20.07**
**Proteases**	**Control**	33.73 ± 1.89	39.97 ± 0.63
	**EN4**	16.46 ± 1.04	7.0 ± 0.50
**‘t’-value**		9.66*	34.46**

Figures are Mean ± Standard Error. Student’s t-test, *P* ≤ 0.05, **Significant at 1% and * Significant at 5%

### Comet assay

**Fig. 4:**
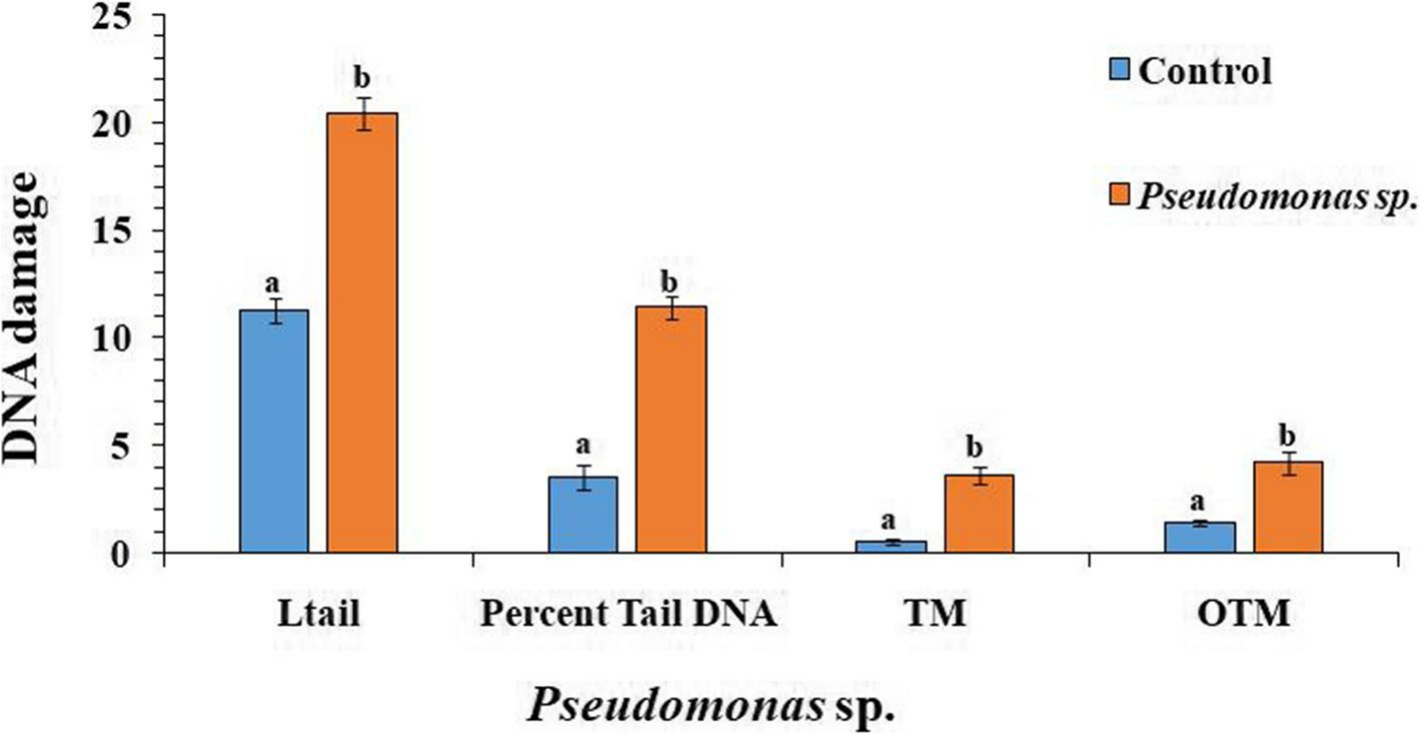
Genotoxic effects of *Pseudomonas* sp. showing variations in DNA damage parameters viz. Tail length (µm) (L tail), Tail DNA (%), Tail moment (TM) and Olive tail moment (OTM) of hemocytes of treated and control *S. litura* larvae. Bars represent the Mean ± SE. Different letters above the bars represent significant differences at Tukey’s test *P* ≤ 0.05

**Fig. 5:**
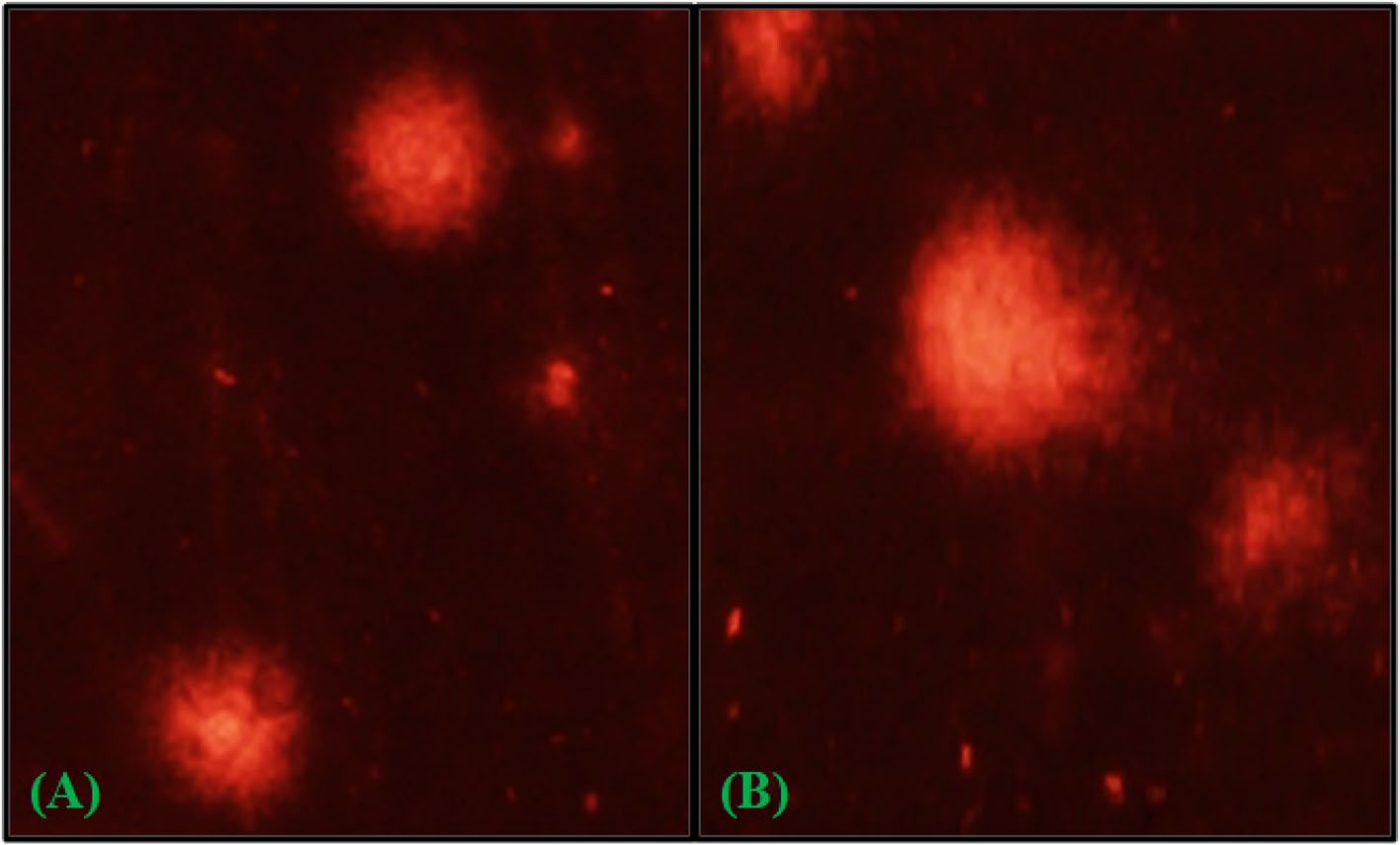
DNA damage in hemocytes of *S. litura* due to bacterial infection. **A** Control, **B** Treatment with *Pseudomonas* sp.

To determine the extent of DNA damage in *S. litura* due to infection of *Pseudomonas* sp., comet parameters viz. tail length, percent tail DNA, tail moment and olive tail moment (OTM) were assessed. The values of all these parameters were significantly higher in larvae fed on bacterial cell suspension for 96 h in comparison to control larvae (Fig. 4). The larvae fed on bacterial-treated leaves showed increased tail length depicting DNA damage in hemocytes as compared to control (Fig. 5). The value of tail length in hemocytes of infected larvae was found to be 20.41 µm compared to 11.23 µm in control (F = 90.34, *p* ≤ 0.05). Similarly, value of percent tail DNA, tail moment and Olive tail moment were found to be 11.42, 3.64 and 4.23 compared to 3.54, 0.53 and 1.39 in control respectively (F = 95.85, *p* ≤ 0.05; F = 60.40, *p* ≤ 0.05; F = 28.12, *p* ≤ 0.05).

## Discussion

There is a growing tendency to identify more pathogenic and effective bacterial biocontrol agents as an effort to develop efficient and eco-friendly methods for controlling insect pests. Thus, in order to search for novel biocontrol agents, different soil bacterial isolates were screened for their insecticidal potential against *S. litura* larvae. Among the tested bacterial isolates, *Pseudomonas* sp., closely related to *P. citronellolis* strain NBRC 103,043 (NR114194) was found to be pathogenic causing 78% larval mortality in *S. litura*. Larvae infected with *Pseudomonas* sp. showed typical symptoms of bacterial infection such as sluggishness, decreased movement, cessation of feeding, flaccid body that turned black and ultimately, death of the larvae. Similar symptoms have earlier been reported in many insect pests due to infection of *B. thuringiensis* and *Pseudomonas aeruginosa* [34, 35].

The presence of bacterial growth in the hemolymph of larvae treated with *Pseudomonas* sp. suggests that larval death occurs due to breaching the gut epithelial barrier and infiltration of bacterial cells in the hemocoel, resulting in septicemia. Similarly, other workers documented mortality in *Spodoptera frugiperda* (J.E. Smith), *S. litura*, *Helicoverpa armigera* (Hubner) due to the proliferation of *Pseudomonas protogens* and *Photorhabdus akhurstii* in hemocoel [36, 37]. The presence of a high bacterial load in the hemolymph causes tissue necrosis as a result of bacterial toxins [38].

The members of genus *Pseudomonas* are widely distributed in the environment and have been isolated most commonly from insect pests and soil samples. A number of *Pseudomonas* species such as *Pseudomonas chlororaphis*, *Pseudomonas taiwanensis*, *Pseudomonas fluorescens, Pseudomonas entomophila*, *Pseudomonas putida*, *Pseudomonas cedrina* and *Pseudomonas paralactis* are known to have insecticidal properties against many insect pests [39–43]. Toxins (Fit toxin, Exotoxin A, ExoS, hydrogen cyanide, rhizotoxins) associated with *P. protogens*, *P. aeruginosa, P. taiwanensis* etc. contribute to pathogenicity by causing sepsis and eventually death of larvae in various insect pests [44–46]. Pathogenicity of *Pseudomonas* species against insects may also be attributed to hydrolytic enzymes such as proteases, chitinases and phospholipases which are known to be produced by these bacterial strains [46, 47]. Metalloproteinases that degrade the internal peptide bonds of proteins inside the gut play a predominant role as a virulence factor of *P. aerugnisosa* [48].

Apart from mortality, larval treatment with *Pseudomonas* sp. extended the overall development period of *S. litura*, decreased adult emergence and induced morphological deformities in adults. Delayed development and reduced adult emergence have earlier been reported in *S. litura*, *H. armigera* and *Drosophila melanogaster* (Meigen) larvae on exposure to *S. marcescens* strain SEN, *B. thuringiensis* and *P. fluorescens* SBW25 [49–51]. The emergence of morphologically deformed adults with underdeveloped and wrinkled wings has been documented previously in *S. litura, Delia radicum* (Linnaeus) and *H. armigera* due to *Bacillus vallismortis*, *Enterobacter cloacae*, *B. thuringiensis* and *P. paralactis* infection [43, 52–54].

The delayed larval development may be correlated with the adverse effect of *Pseudomonas* sp. on nutritional physiology of *S. litura* larvae. Treatment of larval diet with *Pseudomonas* sp. led to a decrease in relative consumption rate with concomitant reduction of the relative growth rate of *S. litura*. There was also a significant reduction in ECI, ECD and AD of *S. litura* when treated with *Pseudomonas* sp. A decrease in ECI value specifies that more food is metabolized for energy and less is converted to body mass (i.e. growth). Food digestibility and the relative amount of food converted to body mass and metabolized for energy needs are the important activities that can affect ECI and ECD values [55]. These studies are in line with the previous findings indicating a decrease in all the nutritional parameters when *S. litura* larvae were treated with bacterial pathogens like *E. cloaca*, *B*. *subtilis*, *B*. *vallismortis*, *Klebsiella pneumoniae* and *P. paralactis* [43, 52, 53, 56].

Histopathological studies indicated extensive damage in the peritrophic membrane and midgut epithelial cells of *S. litura* larvae due to infection of *Pseudomonas* sp. It is well known that the *Pseudomonas* bacteria produce chitinases, which hydrolyze chitin, a common component of the insect exoskeleton and midgut peritrophic membrane [47, 57]. Chitinases have been shown to damage the peritrophic membrane and impair digestion [58]. Delta endotoxin and Vip3Aa toxin of *B. thuringiensis* have been known to damage the epithelial and muscle layers of the gut of lepidopteran insects [59–61]. Similar histological alterations in the midgut have been documented in *S. frugiperda*, *S. litura*, and *H. armigera* larvae following exposure to *P. akhurstii*, *K. pneumoniae*, and *P. paralactis* [37, 43].

Our studies showed a significant decrease in the activity of digestive enzymes such as α-amylases, glucosidases, galactosidases, lipases and proteases of *S. litura* larvae due to infection of *Pseudomonas* sp. The reduction of digestive enzyme activity may be correlated with the histopathological effects produced by this bacterial strain. Zhang et al. [62] reported the significant decrease in protease, amylase and lipase activity due to the destruction of midgut epithelial cells in adult hazelnut weevil due to *S. marcescens* infection. A similar reduction in digestive enzymes was documented by other workers in various insect pests due to infection with *B. thuringiensis* and *Photorhabdus temperata* [63–65]. The midgut is the main site for the synthesis and secretion of digestive enzymes. The destruction of peritrophic membrane and midgut epithelial cells observed in the present study may have impaired the digestive enzyme activity. The suppression of enzyme activity in the treated insects may be due to an imbalance in the enzyme–substrate complex and inhibition of peristaltic movement of the gut thus affecting the efficacy of digestion and nutrient absorption [66].

*Pseudomonas* sp. also caused genotoxicity to *S. litura* as is evident from DNA damage in the hemocytes of *S. litura* larvae. There is very little information available on the genotoxic effects of bacterial biocontrol agents, although there are reports of genotoxicity of plant and fungal extracts to lepidopteran insects [67, 68]. Oberholster et al. [69] observed DNA damage in insects such as *Periplaneta americana* (Linnaeus), *Tenebrio molitor* (Linnaeus) and *Gryllus bimaculatus* (De Geer) caused by a cyanobacterial secondary metabolite (microcystin-LR). Bacterial toxins cause DNA single-strand and double-strand breaks, which activate the classical DNA damage response, resulting in cell cycle arrest or cell death [70, 71]. Due to direct adverse impact on the DNA of hemocytes, the cellular immune system of insect pest may get impaired, and thus rendering it more susceptible to pathogenic infections [72].

For future implications of *Pseudomonas* sp. in integrated pest management (IPM) practices, there is a need to standardize the mass production techniques of bacterial isolate to make it cost effective so that farmers can easily use and further to evaluate its field efficacy. These can also be used in combination with other biological or chemical control agents so as to provide the effective pest control in IPM programs.

## Conclusion

The current work describes the pathogenicity of *Pseudomonas* sp. against *S. litura* larvae. *Pseudomonas* sp. significantly increased the larval mortality rate, delayed the overall development period, decreased adult emergence and induced morphological deformities in adults. The bacterial infection also caused damage to the epithelial membrane and peritrophic matrix of the larval midgut which may further interrupt the digestion ability and nutritional physiology. Insect hemocytes play an essential role throughout the growth and developmental stages of the insects by providing defensive (immune) functions. Genotoxic damage caused by *Pseudomonas* sp. to larval hemocytes of *S. litura* larvae thus may have induced detrimental effects on the insect’s growth and development as observed in the bioassays and made them more susceptible to infection. In conclusion, *Pseudomonas* sp. has the potential to be used as a biocontrol agent against insect pests, however, further research to improve the mass production techniques of bacterial cells and their testing in field conditions is required.

## Materials and methods

### Insect culture

Egg masses and larvae of *S. litura* were collected from cabbage and cauliflower fields near Amritsar (Punjab), India. The larvae were reared in the laboratory on fresh castor leaves in plastic jars (15 cm × 10 cm) at 25 ± 2°C temperature and 65 ± 5% humidity, respectively. Until pupation, the larval diet was changed regularly. Pupae were moved to pupation jars and newly emerged adults were transferred to oviposition jars. Honey solution (1 part honey: 4 parts water v/v) soaked on a cotton swab was given to the adults. The newly hatched larvae were transferred to fresh castor leaves.

### Bacterial cultures

Five bacterial cultures viz. EN1, EN2, AA5, EN4 and R1, were procured from the Department of Microbiology, Guru Nanak Dev University, Amritsar (Punjab), India. All the cultures were isolated from soil samples collected from different locations.

### Maintenance of bacterial cultures

The bacterial cultures (EN1, EN2, AA5, EN4 and R1) were maintained on Luria Bertani (LB) plates. A single colony of each bacterial isolate was inoculated into LB broth and incubated for 48 h at 30°C. To obtain the pellet, each bacterial culture was centrifuged at 10,000 rpm and 4^ο^C for 10 min after incubation. The pellet was resuspended in 1 ml Phosphate Buffer Solution (PBS) (pH 7.0) after being rinsed once with sterile distilled water. The bacterial density was determined at optical density (OD_600_) and adjusted to 1.89 (1.8 × 10^9^ cfu/ml approximately) before being utilized in bioassays as described by Thakur et al. [52].

### Screening assay

Second instar (6 days old) larvae of *S. litura* were used for testing the insecticidal potential of bacterial isolates. The larvae were randomly selected from the lab culture and placed in rearing vials. Fresh castor leaves were surface sterilized with NaOCl (5% v/v), then washed in distilled water. These leaves (about 10 cm^2^) were treated by immersing them in 10 ml bacterial cell suspension. After air drying at room temperature, the treated leaves were placed in rearing containers. Only one larva was kept in a container to avoid cannibalism. The experiment was replicated five times with 50 larvae (10 larvae per replicate) for initial screening. The control group was fed on surface sterilized castor leaves soaked in PBS buffer. The experimental conditions were kept at a constant temperature of 25 ± 2°C and relative humidity of 65 ± 5%. The food was changed after every 48 h and the fresh castor leaves treated with freshly made bacterial suspension were provided to larvae. The mortality of the larvae was monitored daily. The bacterial isolate EN4 exhibiting the highest mortality was selected for detailed studies and identified based on 16s RNA.

### Concentration response test

Based on the highest larval mortality in *S. litura* as per screening test, EN4 was selected for the concentration response test. Five different bacterial concentrations i.e. C1 = 1.2 × 10^3^ cfu/ml, C2 = 1.9 × 10^5^ cfu/ml, C3 = 2.6 × 10^6^ cfu/ml, C4 = 3.4 × 10^7^ cfu/ml, and C5 = 4.3 × 10^9^ cfu/ml (based on their OD_600_ values) were prepared. The castor leaf discs (approximately 10 cm^2^) were treated with ten ml of each concentration. The PBS-dipped leaves were used as control. Experiments were carried out on 50 larvae (6 days old) with 5 replications (10 larvae per replicate) for each concentration. The diet was changed every 48 h till pupation. Daily observations were taken on larval mortality and development. Data was also collected on adult emergence and morphological deformities. Probit analysis using the SPSS 16.0 statistical program was used to determine the lethal concentration (LC_50_) value based on larval mortality data.

### Nutritional analysis

Second instar larvae of *S. litura* were starved for 3–4 h to evaluate the effect of bacterial infection on nutritional physiology. The above-mentioned concentrations were used for this analysis. The larvae were weighed individually and released in rearing vials containing treated leaves of known weight. The leaves dipped in PBS only were used as a control. For each concentration, 25 larvae were used in the experiment (five larvae per replicate). The weight of the larvae, residual diet, and faecal matter was measured after 72 h of feeding, and the overall change in each variable was compared to the previous value. At the end of experiment, the dry weight of larva, residual diet and faecal matter was also recorded by incubating at 60°C for 72 h to determine the loss of water. The data were utilized to generate nutritional indices on a dry weight basis by following the procedure of Farrar et al. [73]:

1 $$RGR=\frac{\mathrm{Change}\;\mathrm{in}\;\mathrm{larval}\;\mathrm{dry}\;\mathrm{weight}/\mathrm{day}}{\mathrm{Starting}\;\mathrm{larval}\;\mathrm{dry}\;\mathrm{weight}}$$

2 $$RCR=\frac{\mathrm{Change}\;\mathrm{in}\;\mathrm{diet}\;\mathrm{dry}\;\mathrm{weight}/\mathrm{day}}{\mathrm{Starting}\;\mathrm{larval}\;\mathrm{dry}\;\mathrm{weight}}$$

3 $$ECI=\frac{\mathrm{Dry}\;\mathrm{weight}\;\mathrm{gain}\;\mathrm{of}\;\mathrm{insect}}{\mathrm{Dry}\;\mathrm{weight}\;\mathrm{of}\;\mathrm{food}\;\mathrm{ingested}}\times100$$

4 $$ECD=\frac{\mathrm{Dry}\;\mathrm{weight}\;\mathrm{gain}\;\mathrm{of}\;\mathrm{insect}}{\mathrm{Dry}\;we\mathrm{ight}\;\mathrm{of}\;\mathrm{food}\;\mathrm{ingested}-\mathrm{Dry}\;\mathrm{weight}\;\mathrm{of}\;\mathrm{frass}}\times100$$

5 $$AD=\frac{\mathrm{Dry}\;\mathrm{weight}\;\mathrm{of}\;\mathrm{food}\;\mathrm{ingested}-\mathrm{Dry}\;\mathrm{weight}\;\mathrm{of}\;\mathrm{frass}}{\mathrm{Dry}\;\mathrm{weight}\;\mathrm{of}\;\mathrm{food}\;\mathrm{ingested}}\times100$$

where, RGR = Relative growth rate, RCR = Relative consumption rate, ECI = Efficiency of conversion of ingested food, ECD = Efficiency of conversion of digested food, AD = Approximate digestibility.

### Histopathological analysis

The effect of EN4 infection was also investigated on the histology of the midgut tissue of *S. litura* larvae. The leaves treated with LC_50_ value of bacterial cell suspension were fed to second instar larvae (6 days old) for 96 h. The larvae that were fed on PBS-treated leaves served as control. The temperature and humidity levels were kept at 25 ± 2^ο^C and 65 ± 5%, respectively. The larvae were dissected aseptically and the larval guts were extracted in distilled water after 96 h. The gut was kept in 10% formalin until the tissue was processed. The sample was washed with distilled water in a tube after fixation, and the process was repeated several times. Dehydration of tissue was achieved by passing it through alcohol concentrations ranging from 30 to 90%. The tissue was fixed in paraffin wax. The microtome was used to make tiny ribbons from wax blocks. These thin ribbons with gut sections were placed on a slide coated with a layer of 1% Mayer's egg albumin and kept warm on a hot plate at 40–45°C to ensure even wax distribution. The slides were rinsed in 100, 90, 80 and 70% ethanol for 1 min each before being dewaxed in xylene for 10 min. The methodology by Dutta et al. [37] was used to stain permanent slides with hematoxylin and eosin stain. Finally, before mounting the samples in Dibutylphthalate Polysterene Xylene (DPX), the slides were rinsed in xylene for 5 min. Images were then captured at 400x magnification using Evos XL core light microscope.

### Assessment for the presence of bacteria in larval hemolymph

The presence of bacteria in the larval hemolymph was determined by feeding second instar larvae on LC_50_ value of EN4. After 96 h of bacterial treatment, 100 µl of hemolymph was collected from ten infected larvae of bacteria-treated groups and ten control larvae. The hemolymph was serially diluted and spread over LB agar plates. Plates were incubated at 30°C for 48 h and the formation of bacterial colonies was observed.

### Biochemical analysis

To study the effect of bacterial infection on digestive enzymes third instar larvae (10 days old) of *S. litura* were fed on leaves treated with LC_50_ concentration of EN4 for 48 and 96 h. The control group larvae were fed on a diet devoid of the bacterial suspension. For each treatment, ten third instar larvae were obtained from each replicate (3 replicates). The larval guts were collected and homogenized (1% gut homogenate) for enzymatic analysis.

### α-Amylases

The activity of α-amylases was determined according to the protocol of Mehrabadi et al. [74]. Enzyme extract (20 µl) was added to tubes containing 100 µl of phosphate buffer (0.02 M) (pH 7.1) and incubated at 35°C for 30 min. The reaction was stopped by adding 100 µl of dinitrosalicylic acid (DNS) reagent by heating it in boiling water for 10 min. The absorbance of the mixture was measured at 540 nm on a microplate reader (Eon BioTek) Winooski, Vermont, USA. Serial dilutions of 0.01 M maltose (100–1000 µM) were used to create the standard curve.

### Glucosidases

The estimation of α glucosidases was carried out following the procedure of Zibaee [75]. Enzyme extract (20 µl) was pre-incubated at 37 °C for 10 min with 40 μl of p-nitrophenyl-α-D-glucopyranoside (pNαG) (5 mM) and 100 μl of phosphate buffer (0.02 M, pH 7.1). Sodium carbonate (1 M) (150 µl) was used to terminate the reaction [76]. The absorbance was measured at 450 nm with a microplate reader (Eon BioTek) at Winooski, Vermont, USA. p-Nitrophenol (0.01 M) was used as standard and concentrations ranging from 100–1000 µM were prepared for constructing a standard curve. A similar procedure was applied in the case of ß glucosidases activity estimation, however, the substrate used in this case was p-nitrophenyl- ß-D-glucopyranoside (pNßG) (5 mM).

### Galactosidases

The activity of α galactosidases was estimated by incubating 20 μl of gut homogenate with 40 μl of p-nitrophenyl- α-D-galactopyranoside (5 mM) and 100 μl of phosphate buffer (0.02 M, pH 7.1) at 37°C for 10 min [75]. The reaction was stopped by adding 150 μl of sodium carbonate (1 M) [76]. The absorbance was recorded on a microplate reader (Eon BioTek) at 450 nm. The standard curve was prepared using serial dilutions of 0.01 M p-nitrophenol (100–1000 µM). Similar procedure was used for the estimation of ß glucosidases activity except for the substrate used in this case was p-nitrophenyl- ß-D- galactopyranoside (pNßG) (5 mM).

### Lipases

The activity of lipases was estimated following the protocol of Tsujita et al. [77]. The enzyme extract (20 µl) and 40 µl of p-nitrophenyl butyrate (27 mM) were added to 100 μl of phosphate buffer (0.02 M, pH 7.1) and incubated at 37°C. After 1 min, 100 μl of NaOH (1 M) was added and absorbance was recorded on a microplate reader (Eon BioTek) at 405 nm. A standard curve was prepared using 100- 1000 µM of p-nitrophenol (0.01 M) and enzyme activity was calculated as µM/mg fresh larval weight.

### Proteases

Protease estimation was done using hemoglobin (20 mg/ml) as substrate according to the protocol of Cohen [78] with slight modifications. Hemoglobin solution (40 μl) was added to 100 μl of phosphate buffer (0.02 M, pH 7.1). The reaction was initiated by adding 40 μl of enzyme extract and incubating at 30°C for 120 min. The reaction was terminated by adding 100 μl of 30% TCA and absorbance was recorded on a microplate reader (Eon BioTek) at 410 nm. Bovine serum albumin (0.01 M) was used as standard and concentrations ranging from (100–1000 µM) were prepared for constructing a standard curve.

### Comet assay

The level of DNA damage was measured using the comet assay. The comet assay was performed in alkaline conditions, with slight modifications, according to Singh et al. [79]. The larvae were fed on castor leaves treated with LC_50_ value of selected bacterial isolate (EN4) (pH 7.4) for 96 h. The prolegs of third instar larvae were shrugged off and the hemolymph (from ten larvae) was collected in eppendorf tubes containing phosphate buffer. The slides were coated with 1% normal melting point agarose (NMPA) and hemocytes were layered on coated slides and kept in a refrigerator at 4ºC to settle down. After that, the slides were soaked in the lysing solution (2.5 M NaCl, 100 mM EDTA, 0.25 M Tris aminomethane, 0.25 M NaOH, 1% Triton X-100, 10% DMSO, double distilled water, pH 10.0) and kept in the refrigerator overnight. Electrophoresis was performed using an electrophoretic unit (25 V; 300 mA) containing electrophoretic buffer (1 mM EDTA, 300 mM NaOH, double distilled water, pH > 13) for 20 min. In a neutralization buffer, the slides were neutralized for 15 min (0.4 M Tris amino methane, double distilled water pH 7.5). After drying the slides were stained with ethidium bromide (50 g/ml) and viewed with a Nikon fluorescence microscope. The experiment was replicated thrice. The tail length, tail moment, percent tail DNA and Olive Tail Moment were calculated using Casplab Software (OTM).

### Statistical analysis

The experiments on larval mortality, development duration, adult emergence, adult deformities and nutritional analysis parameters were replicated five times, while comet and biochemical assays were replicated thrice. The Mean ± SE of all the values was used to represent them. One way analysis of variance (ANOVA) with Tukey’s test at *p* ≤ 0.05 was used to compare the differences in means. Statistical analysis was carried out by using SPSS software for windows version 16.0 (SPSS Inc, Chicago) and Microsoft Office Excel 2007 (Microsoft Corp., USA). For biochemical analysis, paired sample t-test was used.

## Data Availability

The datasets used and/or analysed during the current study is available from corresponding author on reasonable request.
